# Molecular analysis of androgen receptor splice variant AR-V3 reveals eminent ambiguity regarding activity and clinical utility

**DOI:** 10.1186/s12935-025-03948-y

**Published:** 2025-08-26

**Authors:** Neele Wüstmann, Verena Humberg, Julia Vieler, Konstantin Seifert, Martin Bögemann, Katrin Schlack, Andres Jan Schrader, Christof Bernemann

**Affiliations:** Department of Urology, University Hospital Muenster, Albert-Schweitzer Campus 1 A1, 48149 Muenster, Germany

**Keywords:** Androgen receptor, Androgen receptor splice variant 3, Androgen receptor targeted agents, Biomarker, Liquid biopsy, Metastatic castration resistant prostate cancer, Predictive, Prognostic

## Abstract

**Background:**

Androgen receptor (AR) splice variants (AR-Vs) have emerged as potential resistance mechanisms to AR-targeted agents (ARTAs) in prostate cancer (PC), particularly in the context of castration-resistant disease. Among them, AR-V3 has been frequently detected, yet its biological function remains unclear due to conflicting results from initial studies. This study aimed to comprehensively investigate the molecular structure, activity, and clinical relevance of AR-V3.

**Methods:**

We constructed plasmids encoding two AR-V3 isoforms—one newly identified isoform matching the human reference genome (AR-V3^ref^) and the other based on the 22Rv1 cell line sequence (AR-V3^22Rv1^)—and transfected them into AR-negative PC-3 cells alone or co-expressed with AR full-length (AR-FL). Localization, transcriptional activity (via luciferase assays), and RNA sequencing were performed. Protein structure modeling was conducted using AlphaFold2. Nonsense-mediated decay (NMD) was assessed through pharmacological inhibition. Clinically, AR-V3 expression in circulating tumor cells (CTCs) from 65 patients starting ARTA treatment was analyzed in relation to progression-free survival (PFS) and overall survival (OS).

**Results:**

RNA-seq of AR-FL + AR-V3 vs. AR-FL alone showed no AR-V3-specific gene expression. Structure modeling revealed poor overall prediction confidence, particularly in the N-terminal domain, with no consistent structural features differentiating AR-V3^ref^ and AR-V3^22Rv1^. Both isoforms localized mainly to the cytoplasm, regardless of hormonal stimulation or AR-FL co-expression. Neither isoform showed androgen receptor element (ARE) binding activity unless co-expressed with AR-FL. NMD analysis indicated neither isoform was degraded by this pathway. Clinically, AR-V3 + patients had significantly shorter OS (median 13 vs. 23 months; p = 0.02) among CTC + patients but showed no difference in PSA response or PFS under ARTA treatment.

**Conclusions:**

Our data demonstrate that both AR-V3 isoforms are functionally inactive, lacking autonomous nuclear translocation or transcriptional activity. AR-V3 is not a substrate for NMD, and its protein structure remains poorly defined. While associated with worse overall survival, AR-V3 lacks predictive value for ARTA response, underscoring its limited utility as a biomarker. These findings emphasize the need for functional validation before integrating putative biomarkers into clinical practice.

**Supplementary Information:**

The online version contains supplementary material available at 10.1186/s12935-025-03948-y.

## Introduction

Androgen receptor (AR) splice variants (AR-Vs) have been described as a potential mechanism of resistance to AR targeted agents (ARTA), e.g. enzalutamide or abiraterone in prostate cancer (PC), eventually leading to castration resistant prostate cancer (CRPC) [1, 2]. Most AR-Vs lack a functional ligand binding domain (LBD), preceding to either constitutive or conditional active AR variants even in the absence of androgens and vice versa in the presence of LBD targeting agents, e.g. enztamide. Thus, AR-Vs have gained clinical interest as potential predictive biomarker for NHT treatment [alu^3^–^5^].

**Fig. 1:**
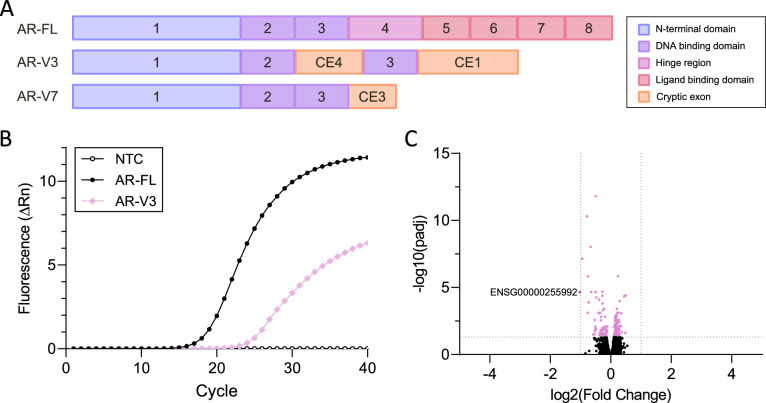
Structure of AR-FL and AR splice variants and RNA sequencing of AR-FL and AR-V3 co-transfected cells **A** Shown are the coding sequences of AR-FL (exon 1–8) as well as AR-V 3 and AR-V7. **B** Amplification curves of PC-3 cells co-transfected with AR-FL and AR-V3. NTC: Non-template control. **C** Volcano Plot of RNA sequencing results of PC3 cells co-transfected with AR-FL and AR-V3 in comparison to AR-FL transfected cells

Among other splice variants, AR-V3 was detected in parallel in three different studies [6–8]. Structurally, AR-V3 contains the coding sequence within exons 1–2-CE4, with CE4 being a cryptic exon located in intron 2. Whereas AR^1/2/2b^ (by Dehm et al.) contains no further exons, AR-V3 (by Hu et al.) and AR6 (by Guo et al.) consist of exons 1–2-CE4-3-CE1 (Fig. 1A). Guo et. al. revealed no transcriptional activity of AR splice variant AR6 in in vitro studies [6]. Hu et al. decided not to pursue any experiments on activity of AR-V3 based on structural peculiarities, i.e. disruption of the DNA binding domain (DBD) by integration of CE4 between exon 2 and exon 3, which, in their opinion, would lead to a non-functional protein [7]. In contrast, Dehm and colleagues demonstrated constitutive activity of AR^1/2/2b^, leading to AR pathway activation in the absence of androgens [8]. Translationally however, both mRNA variants lead to identical proteins with a stop codon located in CE4.

Others and we have demonstrated that AR-V only appear concomitant with high AR-FL expression in clinical samples [3, 5, 9, 10]. Thus, analysis on activity might be more definitive in a setting considering co-expression of both, AR-FL and AR-V instead of sole overexpression of AR-V [8, 9]. Thus, we intended to analyze molecular mechanisms of AR-V3 expression in a more reliable co-expression system. Furthermore, given the discrepancy within the initial studies, we aimed to analyze the molecular structure and genuine activity of AR-V3.

## Results

### Analysis of AR-V3 target genes by RNA sequencing

We performed plasmid-based transfection in AR negative PC-3 cells using an AR-V3 expression plasmid designed according to the human reference genome GRCh38. To mimic the in vivo co-appearance of AR-FL and AR-V3, AR-FL was co-transfected at equimolar concentrations. Besides, sole AR-FL transfection was performed as a control. Analysis of amplification curves by qPCR demonstrated successful transfection of both AR-FL and AR-V3, yet with lower expression levels of AR-V3 compared to AR-FL (Fig. 1B). When running RNA sequencing of AR-FL + AR-V3 in comparison to AR-FL, no obvious differences within gene expression profiles were detected (Fig. 1C). The only differentially expressed gene ENSG00000255992 is an RNA gene encoding PUS1 antisense RNA 1, which belongs to the class of long non-coding RNAs (lncRNA). This suggests lack of AR-V3 specific target genes.

### Identification and molecular analysis of two distinct AR-V3 isoforms

**Fig. 2:**
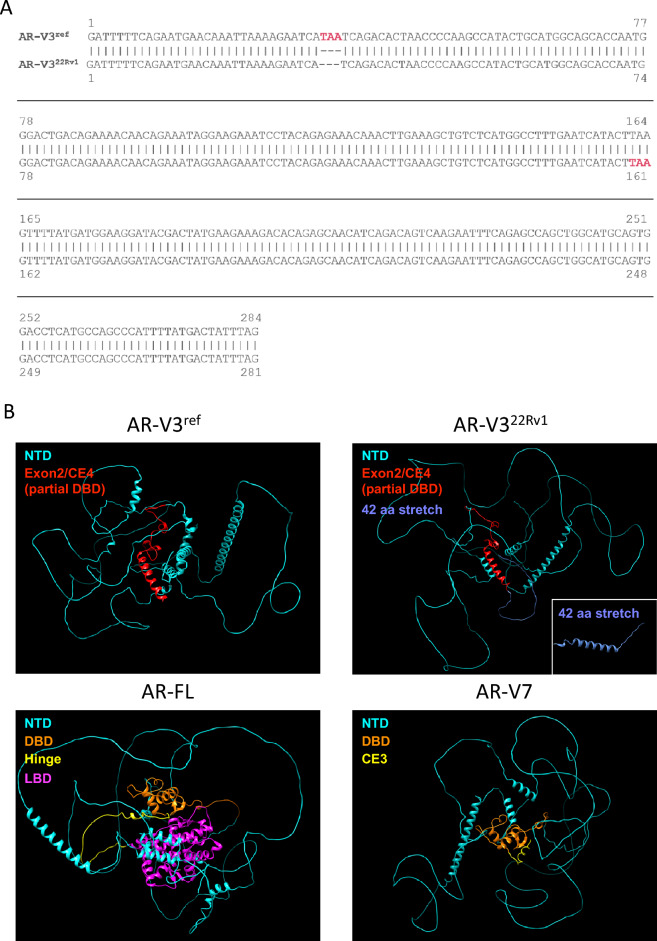
Genomic and structural differences of AR-V3 isoforms **A** Comparison of CE4 DNA sequence of AR-V3^ref^ and AR-V3^22Rv1^. Stop codons are labelled in red. **B** Protein structure models of AR-V3^ref^, AR-V3^22Rv1^ with separately plotted AR-V3^22Rv1^ specific 42 amino acid stretch (inlay), AR-FL and AR-V7. Domains are depicted in respective colors. Models were calculated with AlphaFold2 and visualized with Chimera

These results prompted us to reanalyze the coding sequence of AR-V3. In contrast to Hu et al., we noticed a shorter coding region in CE4. Whereas Hu et al., revealed 158 nucleotides in CE4 before translation termination, we detected a stop codon after 32 nucleotides in CE4 (Fig. S1). This stop codon is equivalent to the human reference genome GRCh38. We next performed sequencing analysis of 22Rv1 cells, in which the initial AR-V3 has been detected [6–8]. We noticed lack of three nucleotides encoding the stop codon within the reference genome, but rather a downstream nucleotide triplet leading to a posterior translation termination signal (Fig. 2A). When analyzing a set of distinct cell lines and PC tissue samples, we observed the prior stop codon in all samples, except 22Rv1 cells (Fig. S2). Thus, we discovered a coding sequence for AR-V3, determined in the reference genome and detected in multiple clinical tissues, of 1803 nucleotides in contrast to the coding sequence of 1929 nucleotides for AR-V3 detected in 22Rv1 cells. We denominated the shorter, yet GRCh38 reference genome version of this AR-V3 isoform AR-V3^ref^ in contrast to AR-V3^22Rv1^.

Subsequently, we performed protein structure modelling of both AR-V3 isoforms using AlphaFold2 (Fig. 2B, S4). Unfortunately, the overall quality of models was low, with a predicted local distance difference test (pLDDT) below 45, revealing very low model confidence. Consequently, structure prediction of uncharacterized regions is limited and hampered by the size of these regions. The NTD of both, AR-V3^ref^ and AR-V3^22Rv1^, displayed dissimilarities, yet with a low quality (predicted local distance difference test (pLDDT) below 50). The partial DBD however, showed similar structures with higher quality (pLDDT of up to more than 90 for α-helices located in the partial DBD). We also run a prediction on the 42 aa stretch specific for AR-V3^22Rv1^. Prediction revealed a possible helical structure of this peptide (pLDDT < 67). However, this helix did not appear within the prediction model for AR-V3^22Rv1^, underlining the high variance of structure prediction for less characterized proteins. Furthermore, we run a prediction on both, AR-FL and AR-V7. Whereas the overall quality was still very low (pLDDT < 55 for AR-FL and < 48 for AR-V7), the two zinc finger containing DBD was detected with high confidence (pLDDT > 90) in both models. Yet, a similar structure was detected neither in the model of AR-V3^ref^ nor AR-V3^22Rv1^. Thus, no structural differences between AR-V3^ref^ and AR-V32^2Rv1^ could be determined.

### Comparison of localization and activity of AR-V3 isoforms

**Fig. 3:**
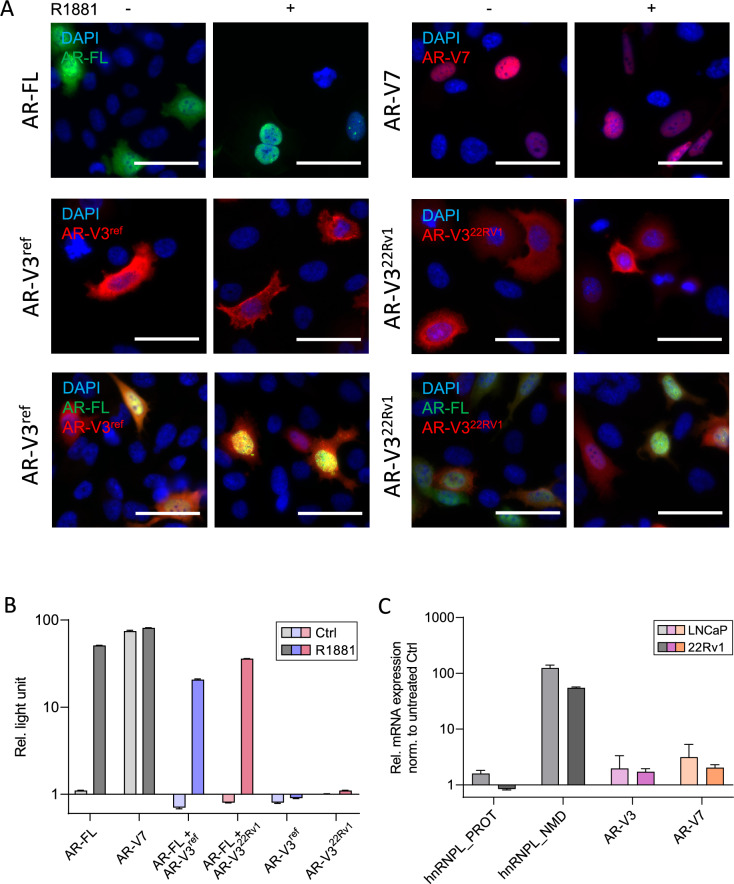
Molecular analysis of AR-V3 isoform localization and protein activity **A** Immunofluorescence staining of PC-3 cells transfected with either AR-FL, AR-V7 (both upper panel), AR-V3^ref^ and AR-V^22Rv1^ (both middle panel) solely or AR-V3 isoforms co-transfected with AR-FL (lower panel) in absence or presence of 1 nM R1881. Bars represent 50 µm. **B** AR activity luciferase assay analysis in indicated single or co-transfected cells. Columns of samples treated with 1 nM R1881 are in darker color shade. **C** NMD assay determination: mRNA expression is shown for hnRNPL_PROT (NMD insensitive isoform), hnRNPL_NMD (NMD sensitive isoform), AR-V3 (AR-V3^ref^ and ARV^22Rv1^ for LNCaP and 22Rv1 respectively) and AR-V7 in LNCaP and 22Rv1 cells treated with 1 µM hSMG-1 inhibitor 11e. LNCaP samples are represented in a light color shade and 22Rv1 samples in darker shades. NMD: nonsense-mediated decay

We next designed a plasmid containing the coding region of AR-V3^22Rv1^ lacking the prior stop codon to compare with AR-V3^ref^ for localization and activity. Both AR-V3 variants were either transfected alone or in combination with AR-FL into PC-3 cells. AR-FL and AR-V7 transfections were performed as controls for either hormonal dependent activity (AR-FL) or constitutive activity (AR-V7) of AR variants. All transfections were executed in the absence or presence of 1 nM R1881. Whereas AR-FL was mainly detected in the cytoplasm in medium without R1881 addition, supplementation led to nuclear localization of AR-FL (Fig. 3A). AR-V7 was detected in the nucleus irrespective of hormonal stimulation. In contrast, AR-V3^ref^ as well as AR-V3^22Rv1^ were detected mainly in the cytoplasm, regardless of both R1881 supplementation and co-transfection with AR-FL.

We also performed analysis of androgen receptor responsive elements (ARE) binding capacity of both AR-V3 isoforms using luciferase-based assays. Increase of luciferase activity was observed for AR-FL in the presence of R1881, demonstrating dependence on hormonal stimulation to impact ARE binding (Fig. 3B). AR-V7 displayed constitutive activity by similarly high luciferase activities in the absence and presence of R1881. When AR-FL was co-transfected with either AR-V3^ref^ or AR-V3^22Rv1^ we also noticed an increase in luciferase activity, similar to AR-FL transfection. When transfected separately, neither AR-V3^ref^ nor AR-V3^22Rv1^ display significant activity, irrespective of R1881 stimulation. This indicates no independent ARE-binding capacity of both AR-V3 isoforms.

### Analysis of nonsense mediated RNA decay (NMD) of AR-V3 isoforms

Given the specific exon structure of AR-V3 containing two additional exons after termination signal, we performed analysis of whether AR-V3 isoforms might be degraded by nonsense-mediated RNA decay (NMD) [11]. LNCaP and 22Rv1 cell lines, expressing AR-V3^ref^ or AR-V3^22Rv1^, respectively, were used. Deactivation of the NMD pathway by SMG1 inhibition led to increase in NMD sensitive (hnRNPL_NMD) transcripts, whereas NMD insensitive (hnRNPL_PROT) transcripts were not affected by treatment, demonstrating activity of NMD pathway in these cell lines [12]. No changes in mRNA levels were detected for both AR-V3 isoforms. This validates that both AR-V3 isoforms are no substrates for NMD.

### AR-V3 expression and clinical outcome in a cohort of 65 patients undergoing ARTA

**Fig. 4:**
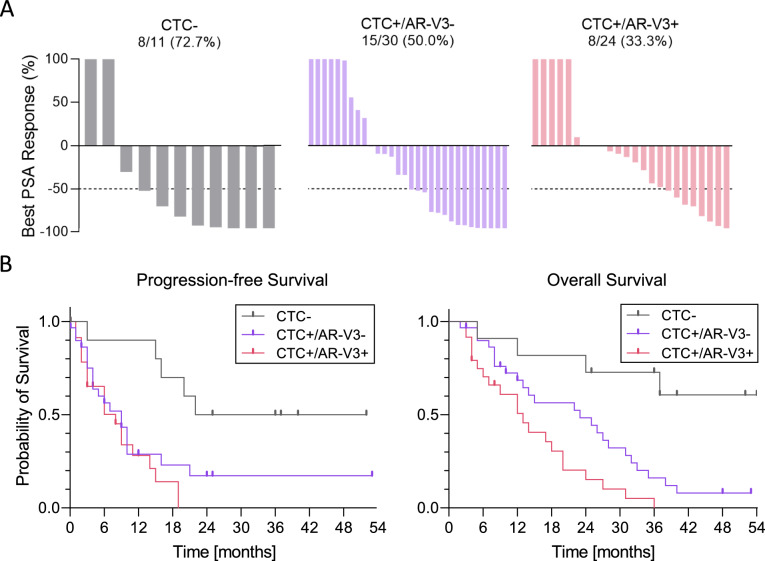
Detection and clinical analysis of AR-V3 in 65 mCRPC patients undergoing ARTA treatment. **A** Waterfall plots depicting best PSA responses in patients according to CTC and AR-V status: CTC- (left panel), CTC +/AR-V3- (middle panel), CTC +/AR-V3 + (right panel). The dotted line illustrates the threshold of PSA 50% decline defining a PSA response. Numbers indicate responding patients including percentages. **B** Kaplan–Meier curves indicating PFS (left panel) and OS (right panel) according to CTC and AR-V3 status. PFS and OS is significantly different between CTC-, CTC +/AR-V3- and CTC +/AR-V3 + (p < 0.01; p < 0.001). Regarding AR-V3 status, CTC +/AR-V3 + patients have a similar PFS than CTC +/AR-V3- patients (p = 0.32) whereas OS is significantly worse for CTC +/AR-V3 + patients (p = 0.02). OS: Overall survival, PFS: Progression-free survival

Finally, a subgroup of patients (n = 65 patients) was comprehensively analyzed for presence of circulating tumor cells (CTCs) and AR-V3 expression in CTCs (irrespective of isoform) before starting ARTA treatment (abiraterone = 46, enzalutamide = 19). At the time of study closure, median follow-up time was 14 (IQR 8–31) months. Baseline characteristics are presented in table S1. Eleven patients (16.9%) were CTC-, 30 (46.2%) CTC +/AR-V3- and 24 (36.9%) CTC +/AR-V3 +. We observed a PSA response in 31 (47.7%) patients. CTC- patients showed a PSA response in eight (72.7%) cases, CTC +/AR-V3- patients in 15 (50.0%) and CTC +/AR-V3 + patients in 8 (33.3%) cases (Fig. 4A).

For the overall cohort, median PFS was 9 months (CI 7.1–10.9), within the three subgroups it was not reached for CTC- patients, 9 months (CI 4.5–13.5) for CTC +/AR-V3- patients and 8 months (CI 3.9–12.1) for CTC +/AR-V3 + patients (p < 0.01) (Fig. 4B left panel). No statistically significant differences could be observed between the CTC +/AR-V3- and CTC +/AR-V3 + subgroups (p = 0.31).

Median OS for the overall cohort was 20 months (CI 10.5–29.5), within the three subgroups it was not reached for CTC- patients, 23 months (CI 7.2–38.8) for CTC +/AR-V3- patients and 13 months (CI 7.8–18.2) for CTC +/AR-V3 + patients (p < 0.01) (Fig. 6B right panel). When analyzing the CTC + subgroups, we noticed a significantly worse OS in CTC +/AR-V3 + patients compared to CTC +/AR-V3- patients (p = 0.02). These results suggest prognostic, yet not predictive property of AR-V3.

## Discussion

Aim of our study was the molecular analysis of AR-V3, a prostate cancer related, presumably clinically relevant AR splice variant. Whereas previous studies delineate expression of AR-V3 as a potential biomarker in the context of ARTA resistance, our results question the genuine role of AR-V3 as a tumor-promoting resistance mechanism in prostate cancer and thus, its value as a clinical biomarker.

During the last decade, AR-Vs have gained clinical interest as potential biomarker in prostate cancer. The most often studied AR splice variant is AR-V7, which has been discussed as both a predictive biomarker for non-response to ARTA treatment and a prognostic biomarker for an advanced stage of disease [13–15]. Besides AR-V7, other AR splice variants have gained clinical interest, e.g., AR-V3, AR-V9 and AR-V12 (a.k.a. AR-V567es) [16–18]. We have previously shown that AR-V567es, which, in contrast to most other AR-Vs, relies on genomic alteration rather than alternative splicing, tends to be detected disproportionately, presumably due to the usage of deficient primers detecting false-positively AR-FL in the absence of AR-V567es [19]. Indeed, we were able to detect AR-V567es in one out of more than 200 clinical specimen only, which is in stark contrast to studies demonstrating appearance of AR-V567es in up to 70% of prostate cancer patients [20, 21].

Likewise, AR-V3 has been described as a clinically relevant resistance mechanism demonstrating lower OS probability in AR-V3 mRNA positive patients compared to AR-V3 mRNA negative patients [22]. AR-V3 exhibits a different structure compared to most other AR splice variants: whereas most AR-Vs contain exons 2 and 3 followed by cryptic exons, AR-V3 contains a cryptic exon CE4 spliced right to exon 2. Thereby, the canonical structure of the two zinc-finger containing DBD encoded by exons 2 and 3 is disrupted. Whereas one of the initial studies demonstrated constitutive activity of AR-V3, another study hypothesized AR-V3 being inactive because of the disrupted DBD, yet without experimental validation [7, 8]. The third study performed activity analysis; however, they could not reveal activity of AR-V3 [6]. The authors hypothesized technical approaches being the reason for discrepant results. Irrespective of these discordant results, further clinical studies implemented analysis of AR-V3 as a potential predictive biomarker for non-response to ARTA treatment in PC patients [5, 9, 23].

During our analysis, we noticed genomic sequence differences between 22Rv1 cells and other clinical samples in terms of the coding region for AR-V3. Whereas 22Rv1 cells, in which the initial AR splice variant AR-V3 (a.k.a. AR^1/2/2b^ or AR6) has been identified, lack a stop codon within the first third of CE4, all other samples analyzed display this stop codon, which is also present in the human reference genome GRCh38. The lack of this premature stop codon leads to a longer protein sequence in 22Rv1, i.e. AR-V3^22Rv1^. We now systematically compare both isoforms, i.e., AR-V3^ref^ and AR-V3^22Rv1^, in a set of molecular explorations. In silico prediction revealed no differences, yet predicted models lack high overall quality, which can be explained by the lack of crystal or cryo-EM structures covering AR-FL or AR-V isoforms or the intrinsically disorder of the AR NTD, complicating valid in silico prediction [24, 25]. In addition, no variances could be observed in terms of nuclear localization or ARE binding activity for both AR-V3 isoforms. These results question the mechanistic role of AR-V3 isoforms as resistance mechanism to ARTA treatment. One explanation might rely on the atypical structure of AR-V3; two additional exon elements are located 3'to the termination signal in CE4; at least in AR-V3 isoforms containing exon 3 and CE1 as part of their mRNA. Thus, the AR-V3 mRNA contains a premature termination codon (PTC) in CE4 followed by two exon elements. This structure might form a substrate for cellular nonsense-mediated RNA decay [26]. However, NMD pathway inhibition did not lead to an increase in mRNA levels of AR-V3^ref^ in LNCaP cells, AR-V3^22Rv1^ in 22Rv1 cells or AR-V7, the latter known not to be a substrate for NMD [27]. Thus, lack of functional activity of AR-V3 isoforms does not rely on RNA decay mechanisms. Whether post-translational mechanisms, e.g. phosphorylation or ubiquitylation, are involved in degradation of AR-V3 isoforms has yet to be determined [28, 29].

Lastly, from a clinical perspective, we revealed no predictive value of AR-V3 in PC patients undergoing ARTA treatment, clearly demonstrating ineffectiveness of AR-V3 as a biomarker. In terms of overall survival, we noticed a significantly worse OS in AR-V3 + patients. Given however, the appearance of AR splice variants at later stages of disease, the presence of AR-V3 in a prognostic worse subgroup is reasonable, yet without additional value [3, 15].

A limitation of our study is the lack of a functional AR-V3^ref^ or ARV3^22Rv1^ antibody. Thus, we are not able to determine protein levels of AR-V3 isoforms in PC specimen. However, our results in terms of localization, ARE binding activity, lack of specific target gene expression along with the deficiency of predictive value for ARTA treatment, indicate no clinical relevance of AR-V3 isoforms.

Overall, AR-V3^ref^ as well as the 22Rv1 cell line specific isoform AR-V3^22Rv1^ must be considered as being functionally inactive and as such, are not eligible as biomarker in PC. Studies, in which AR-V3 has been described as a constitutive biomarker mainly rely on mRNA detection, yet without functional protein translation. Thus, clinical relevance of these findings has to be questioned. Thus, our results demonstrate inevitability of appropriate validation of preclinically generated results before transfer into clinical analyses.

## Methods

### Cell culture

Human cell lines (LNCaP, 22Rv1 and PC-3) were purchased from the Leibniz-Institute DSMZ GmbH (Braunschweig, Germany) and cultured under matching protocols at 37 °C and 5% CO_2_. Media was purchased from Sigma Aldrich (Pasching, Germany). Trypsin–EDTA, phosphate-buffered saline and fetal calf serum were purchased from Thermo Fisher Scientific (Waltham, MA, USA). To mimic different treatment conditions (androgen deprivation or stimulation), PC-3 cells were cultured in combinations of presence and absence of 1 nM androgen analogon R1881 (Sigma Aldrich) and 10 µM enzalutamide (Selleck Chemicals LLC, Houston, TX, USA). For testing NMD activity, LNCaP and 22Rv1 cells were treated with 1 µM hSMG-1 inhibitor 11e for 24 h (Med Chem Express, Monmouth Junction, NJ, USA).

### AR-FL and AR-V3 transfection of cells

AR-FL and AR-V7 plasmids containing either the coding regions of exons 1–8 along or the coding regions of exon 1, exon 2, exon 3 and cryptic exon 3, respectively, with either an eGFP flag or a 3XFLAG tag and the GFP tag AR-V7 plasmid were kindly provided by Dr. Santer (Department of Urology, Medical University of Innsbruck, Austria; GFP constructs) and Dr. Plymate (Department of Medicine, University of Washington School of Medicine, WA, USA, 3XFLAG-tag constructs). A plasmid containing the coding sequence of AR-V3 according to the GRCh38 reference genome was designed and ordered (AR-V3^ref^). A second plasmid including the coding sequencing for AR-V3^22Rv1^ was designed and ordered. This plasmid lacks the GRCh38 termination signal of AR-V3^ref^, but rather the 22Rv1 downstream stop codon. The coding sequences of both plasmids were subcloned into N-terminal GFP protein or N-terminal 3XFlag-tag overexpression vectors. 5 × 10^4^ cells in a 24-well plate were transfected with total amount of 500ng DNA using ViaFect™ Transfection Reagent (Promega, Madison, WI, USA). RNA was isolated 48 h post transfection.

### RNA Seq

Library preparation of total RNA was performed with the NEB Next Ultra II RNA directional Kit and single read sequencing was performed using a NextSeqR® 2000 System with a read length of 72 bp. Using a molecular barcode, the samples were demultiplexed (bcl2fastq2) to fastq data and quality controlled (FastQC). Trimmomatic was used for adapter trimming and read filtering [30]. The resulting reads were aligned to the Ensembl GRCh38 reference genome using Hisat2 [31]. The aligned reads were sorted using samtools [32]. The sorted and aligned reads were counted into genes using htsec-counts [33]. The test for differential expression were performed using the r-package deseq2 [34]. Volcano plots were drawn using Prism 8 V8.4.3 (GraphPad Software, LLC., San Diego, CA, USA).

### qPCR

Total RNA of cell lines was isolated using the RNeasy® Mini kit (Qiagen, Hilden, Germany) according to the manufactures guide. 500 ng of total RNA were reverse transcribed using the Primescript® Reverse Transcription Kit (Takara, Tokyo, Japan). AR-FL expression was analyzed using TaqMan PCR assay (Hs00171172_m1) (Thermo Fisher Scientific). AR-V expression was analyzed by using previously described custom-made TaqMan PCR assays specific for detection of AR-Vs AR-V3 and AR-V7 [19]. Assay sequences are listed in Table S2. All qPCR runs were performed along with TaqMan PCR assays for housekeeping genes RPL37A (Hs01102345_m1) and HPRT1 (Hs99999909_m1) (Thermo Fisher Scientific). Due to relative short 3’ sequences specific for AR-V3 (32 nts for AR-V3^ref^ and 158 nts for AR-V3^22Rv1^), a qPCR assay consisting of a forward primer (spanning exon2—CE4 junction) and a reverse primer located within the plasmid backbone 3’ of AR-V3 isoforms coding regions, was used for detection of transfected transcripts.

For NMD analysis, qPCR analysis on endogenous AR-V3 (either AR-V3^ref^ in LNCaP or AR-V3^22rv1^ in 22Rv1 cells) was performed along with analysis of two hnRNPL transcripts. hnRNPL (heterogeneous nuclear ribonucleoprotein L) is a protein known to be a substrate for NMD by alternative splicing of a short exon within intron 6, which contains a premature stop codon [12]. We used hnRNPL_NMD (a NMD sensitive transcript) and hnRNPL_PRΟΤ (a NMD insensitive transcript) [11]. qPCR reactions for determination of AR-V3 isoform transfection and hnRNPL transcripts were performed using SYBR Green Mastermix along with RPL37A and ACTB assays as housekeeping genes. qPCR reactions were run using the TaqMan Fast Advanced Mastermix on a QuantStudio 3 qPCR cycler (Thermo Fisher Scientific).

### CE4 sequencing analysis

PCR primers spanning the CE4 region containing the reference stop codon were used on genomic DNA of cell lines or clinical tissue samples. Primer sequences are listed in Table S2. PCR amplicons were sanger sequenced and aligned to the reference genome sequence.

### Immunofluorescence

For immunofluorescence analysis, cells were fixated with 4% formaldehyde (Sigma-Aldrich) in DPBS and permeabilized using 1% Triton-X (Sigma-Aldrich) in PBS. Blocking was performed using 1% BSA (Sigma-Aldrich) in PBS. Primary FLAG tag antibody was anti-FLAG (DXKDDDDK tag monoclonal antibody, clone 2B3C4, 1:400, Proteintech, Rosemont, IL, USA) along with a secondary Alexa Fluor™ 546 conjugated antibody (1:500, Thermo Fisher Scientific). DAPI (Sigma-Aldrich) was used as DNA staining solution.

### Androgen receptor responsive element (ARE) binding activity Luciferase assays

For determination of AR activity, cells were co-transfected with an androgen receptor responsive element (ARE) firefly luciferase reporter plasmid (pGL3-4xARE-E4-luc, a gift from Dr. M Carey, Department of Biological Chemistry, UCLA, Los Angeles, CA, USA) and the Renilla luciferase control plasmid pRL-TK (Promega, Madison, WI, USA). Cells were cultured in absence or presence of both 1 nM R1881 and 10 nM enzalutamide along with a DMSO control for 48h. Dual luciferase reporter assays were performed according to the manufacturer’s protocol using the Dual-Glo® Luciferase Assay System (Promega) on a Varioskan Lux microplate reader (Thermo Fisher Scientific). Firefly luciferase activity was normalized to Renilla luciferase activity. Transcriptional activation of Firefly luciferase reporter in R1881 treated cells was presented as luciferase activity relative to the DMSO control.

### Protein structure determination

Protein structure prediction was performed using AlphaFold2 via the Neurosnap platform (Neurosnap Inc.—Computational Biology Platform for Research. Wilmington, DE, 2022. https://neurosnap.ai/) [35, 36]. Sources for protein prediction were amino acid (aa) sequences of AR-FL (NM_000044.6), AR-V7 (NM_001348061.1) and AR-V^22Rv1^ (FJ235920.1) and the 42 aa stretch specific for AR-V3^22Rv1^. AR-V3^ref^ coding region did not align perfectly to any transcript by NCBI BLAST but rather to the genomic sequence NG_009014.2 of the AR gene on chromosome X. Results display 5 prediction models for each sequence in descending order of pLDDT (predicted local distance difference test). For each variant, protein structure model with the highest pLDDT score was visualized using Chimera [37].

### Patient samples and ethics

Analysis of patient blood samples was approved by the local Ethics committee (Ethik-Kommission der Ärztekammer Westfalen-Lippe und der Universität Münster; 2007–467-f-S a) and all patients provided written informed consent. The study was conducted with provisions of the Declaration of Helsinki. Patient characteristics, detection of circulating tumor cells, AR-FL and AR-V3, analyses on PSA response and survival were performed as being described previously [3, 15]

### Statistical analyses

The statistical assessment was performed using R software (version 4.1.3; R Foundation), SPSS-Statistics V25.0 (IBM Inc., Armonk, NY, USA) and Prism 8 V8.4.3. Time-to-event outcomes (PFS and OS) were evaluated performing Kaplan–Meier analysis.

## Supplementary Information

Additional file 1

Additional file 2

Additional file 3

Additional file 4

Additional file 5

Additional file 6

## Data Availability

No datasets were generated or analysed during the current study.

## Abbreviations

ARE: Androgen receptor response element
AR: Androgen receptor
AR-FL: Full length androgen receptor
ARTA: Androgen receptor targeted agents
AR-V: Androgen receptor splice variant
CE: Cryptic exon
CI: Confidence interval
CRPC: Castration resistant prostate cancer
CTC: Circulating tumor cell
DBD: DNA binding domain
GRCh38: Genome reference consortium human build 38
hSMG-1: Human suppressor with morphogenetic effect on genitalia-1
hnRNPL: Heterogeneous nuclear ribonucleoprotein L
IQR: Interquartile range
lncRNA: Long non-coding RNA
LBD: Ligand Bbnding domain
NMD: Nonsense mediated decay
NTD: N-terminal domain
OS: Overall survival
PC: Prostate cancer
PFS: Progression free survival
pLDDT: Predicted local distance difference test (AlphaFold confidence score)
PSA: Prostate specific antigen
RNA-Seq: RNA sequencing
