# Supplementary material for: Molecular analysis of androgen receptor splice variant AR-V3 reveals eminent ambiguity regarding activity and clinical utility
Source: Cancer Cell Int. 2025 Aug 26;25:316. doi: 10.1186/s12935-025-03948-y (PMC12379386; doi:10.1186/s12935-025-03948-y)
Supplement: Supplementary file 1 — Additional file 1 [file 12935_2025_3948_MOESM1_ESM.pptx]

## Slide 1
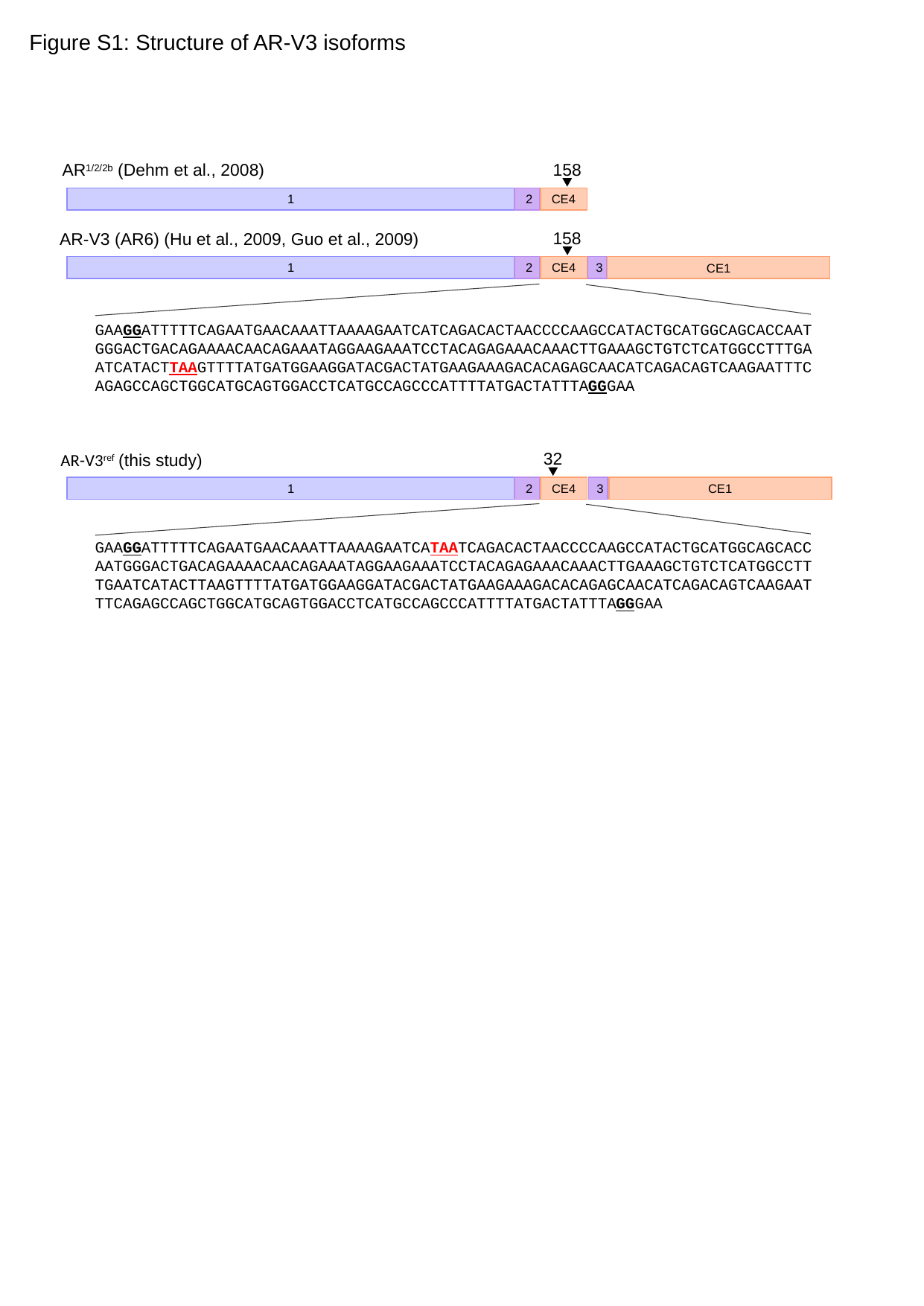

Figure S1: Structure of AR-V3 isoforms
158
AR1/2/2b (Dehm et al., 2008)
CE4
1
2
158
AR-V3 (AR6) (Hu et al., 2009, Guo et al., 2009)
CE4
3
1
2
CE1
GAAGGATTTTTCAGAATGAACAAATTAAAAGAATCATCAGACACTAACCCCAAGCCATACTGCATGGCAGCACCAATGGGACTGACAGAAAACAACAGAAATAGGAAGAAATCCTACAGAGAAACAAACTTGAAAGCTGTCTCATGGCCTTTGAATCATACTTAAGTTTTATGATGGAAGGATACGACTATGAAGAAAGACACAGAGCAACATCAGACAGTCAAGAATTTCAGAGCCAGCTGGCATGCAGTGGACCTCATGCCAGCCCATTTTATGACTATTTAGGGAA
32
AR-V3ref (this study)
1
2
CE4
3
CE1
GAAGGATTTTTCAGAATGAACAAATTAAAAGAATCATAATCAGACACTAACCCCAAGCCATACTGCATGGCAGCACCAATGGGACTGACAGAAAACAACAGAAATAGGAAGAAATCCTACAGAGAAACAAACTTGAAAGCTGTCTCATGGCCTTTGAATCATACTTAAGTTTTATGATGGAAGGATACGACTATGAAGAAAGACACAGAGCAACATCAGACAGTCAAGAATTTCAGAGCCAGCTGGCATGCAGTGGACCTCATGCCAGCCCATTTTATGACTATTTAGGGAA
