# Supplementary material for: Molecular analysis of androgen receptor splice variant AR-V3 reveals eminent ambiguity regarding activity and clinical utility
Source: Cancer Cell Int. 2025 Aug 26;25:316. doi: 10.1186/s12935-025-03948-y (PMC12379386; doi:10.1186/s12935-025-03948-y)
Supplement: Supplementary file 2 — Additional file 2 [file 12935_2025_3948_MOESM2_ESM.pptx]

## Slide 1
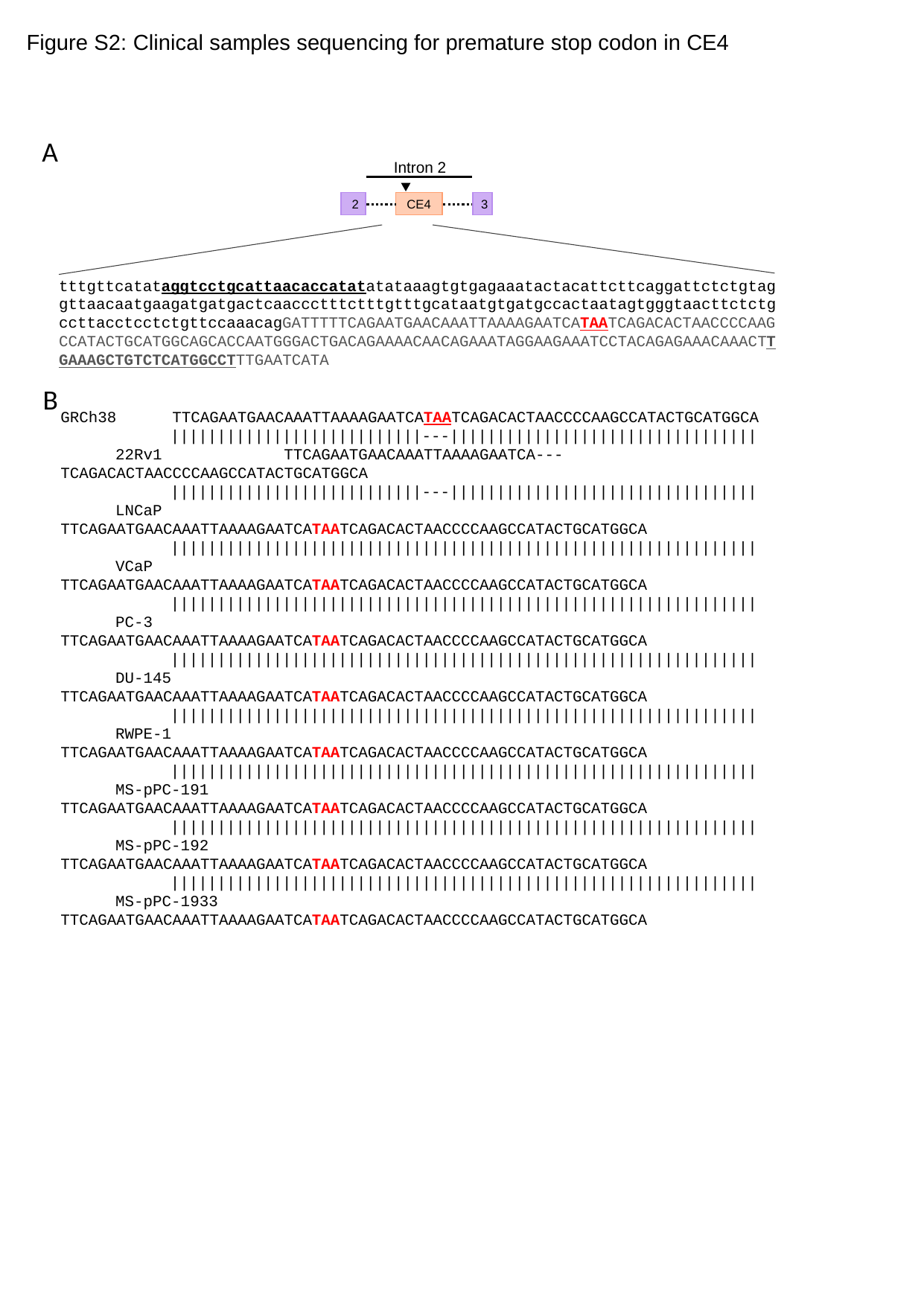

Figure S2: Clinical samples sequencing for premature stop codon in CE4
A
Intron 2
CE4
2
3
tttgttcatataggtcctgcattaacaccatatatataaagtgtgagaaatactacattcttcaggattctctgtaggttaacaatgaagatgatgactcaaccctttctttgtttgcataatgtgatgccactaatagtgggtaacttctctgccttacctcctctgttccaaacagGATTTTTCAGAATGAACAAATTAAAAGAATCATAATCAGACACTAACCCCAAGCCATACTGCATGGCAGCACCAATGGGACTGACAGAAAACAACAGAAATAGGAAGAAATCCTACAGAGAAACAAACTTGAAAGCTGTCTCATGGCCTTTGAATCATA
B
GRCh38	TTCAGAATGAACAAATTAAAAGAATCATAATCAGACACTAACCCCAAGCCATACTGCATGGCA
|||||||||||||||||||||||||||---|||||||||||||||||||||||||||||||||
22Rv1		TTCAGAATGAACAAATTAAAAGAATCA---TCAGACACTAACCCCAAGCCATACTGCATGGCA
|||||||||||||||||||||||||||---|||||||||||||||||||||||||||||||||
LNCaP		TTCAGAATGAACAAATTAAAAGAATCATAATCAGACACTAACCCCAAGCCATACTGCATGGCA
|||||||||||||||||||||||||||||||||||||||||||||||||||||||||||||||
VCaP 		TTCAGAATGAACAAATTAAAAGAATCATAATCAGACACTAACCCCAAGCCATACTGCATGGCA
|||||||||||||||||||||||||||||||||||||||||||||||||||||||||||||||
PC-3 		TTCAGAATGAACAAATTAAAAGAATCATAATCAGACACTAACCCCAAGCCATACTGCATGGCA
|||||||||||||||||||||||||||||||||||||||||||||||||||||||||||||||
DU-145 	TTCAGAATGAACAAATTAAAAGAATCATAATCAGACACTAACCCCAAGCCATACTGCATGGCA
|||||||||||||||||||||||||||||||||||||||||||||||||||||||||||||||
RWPE-1 	TTCAGAATGAACAAATTAAAAGAATCATAATCAGACACTAACCCCAAGCCATACTGCATGGCA
|||||||||||||||||||||||||||||||||||||||||||||||||||||||||||||||
MS-pPC-191	TTCAGAATGAACAAATTAAAAGAATCATAATCAGACACTAACCCCAAGCCATACTGCATGGCA
|||||||||||||||||||||||||||||||||||||||||||||||||||||||||||||||
MS-pPC-192	TTCAGAATGAACAAATTAAAAGAATCATAATCAGACACTAACCCCAAGCCATACTGCATGGCA
|||||||||||||||||||||||||||||||||||||||||||||||||||||||||||||||
MS-pPC-1933	TTCAGAATGAACAAATTAAAAGAATCATAATCAGACACTAACCCCAAGCCATACTGCATGGCA
