# Supplementary material for: Molecular analysis of androgen receptor splice variant AR-V3 reveals eminent ambiguity regarding activity and clinical utility
Source: Cancer Cell Int. 2025 Aug 26;25:316. doi: 10.1186/s12935-025-03948-y (PMC12379386; doi:10.1186/s12935-025-03948-y)
Supplement: Supplementary file 3 — Additional file 3 [file 12935_2025_3948_MOESM3_ESM.pptx]

## Slide 1
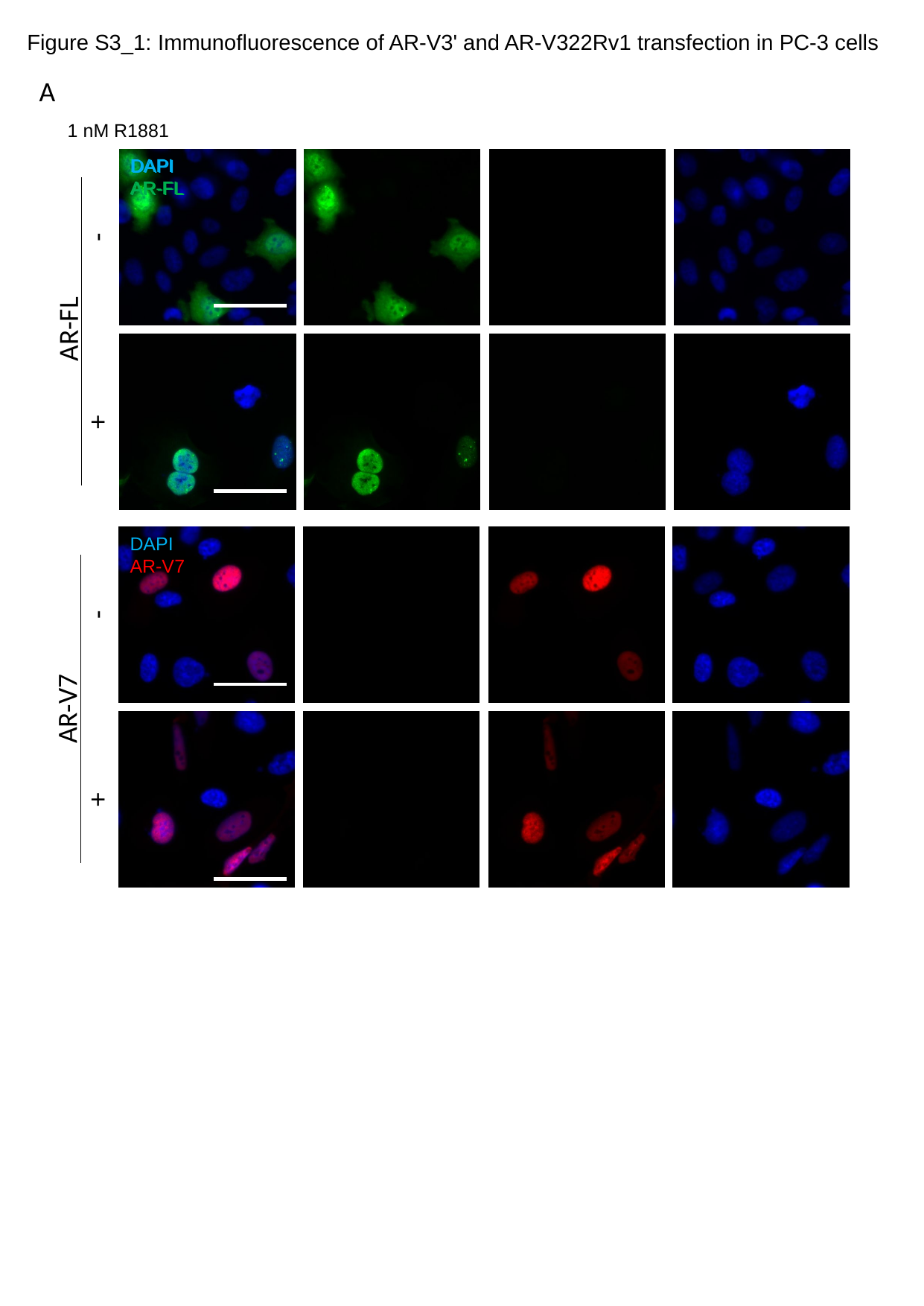

Figure S3_1: Immunofluorescence of AR-V3' and AR-V322Rv1 transfection in PC-3 cells
A
1 nM R1881
DAPI
AR-FL
DAPI
AR-FL
-
AR-FL
+
DAPI
AR-V7
-
AR-V7
+

## Slide 2
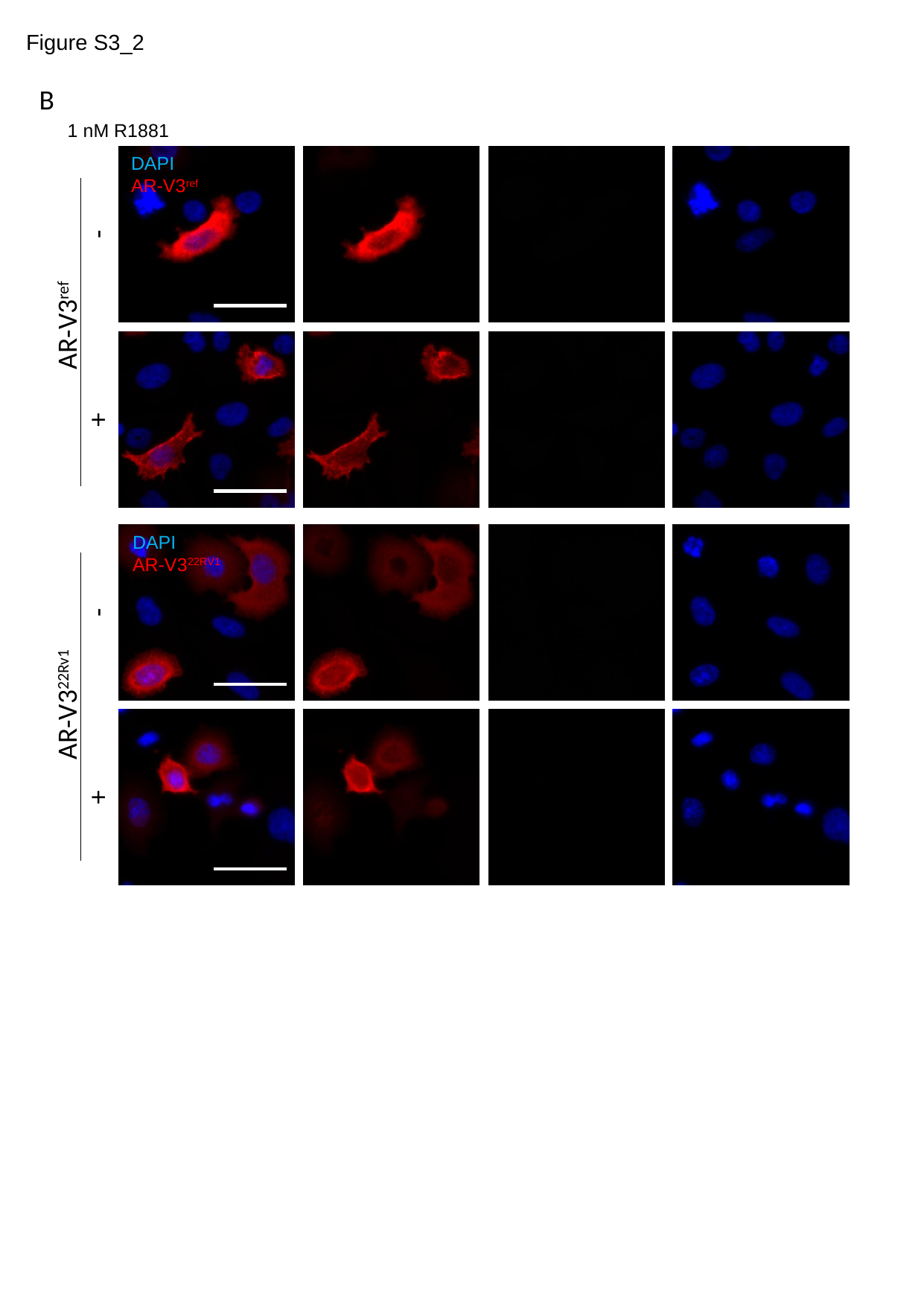

Figure S3_2
B
1 nM R1881
DAPI
AR-V3ref
-
AR-V3ref
+
DAPI
AR-V322RV1
-
AR-V322Rv1
+

## Slide 3
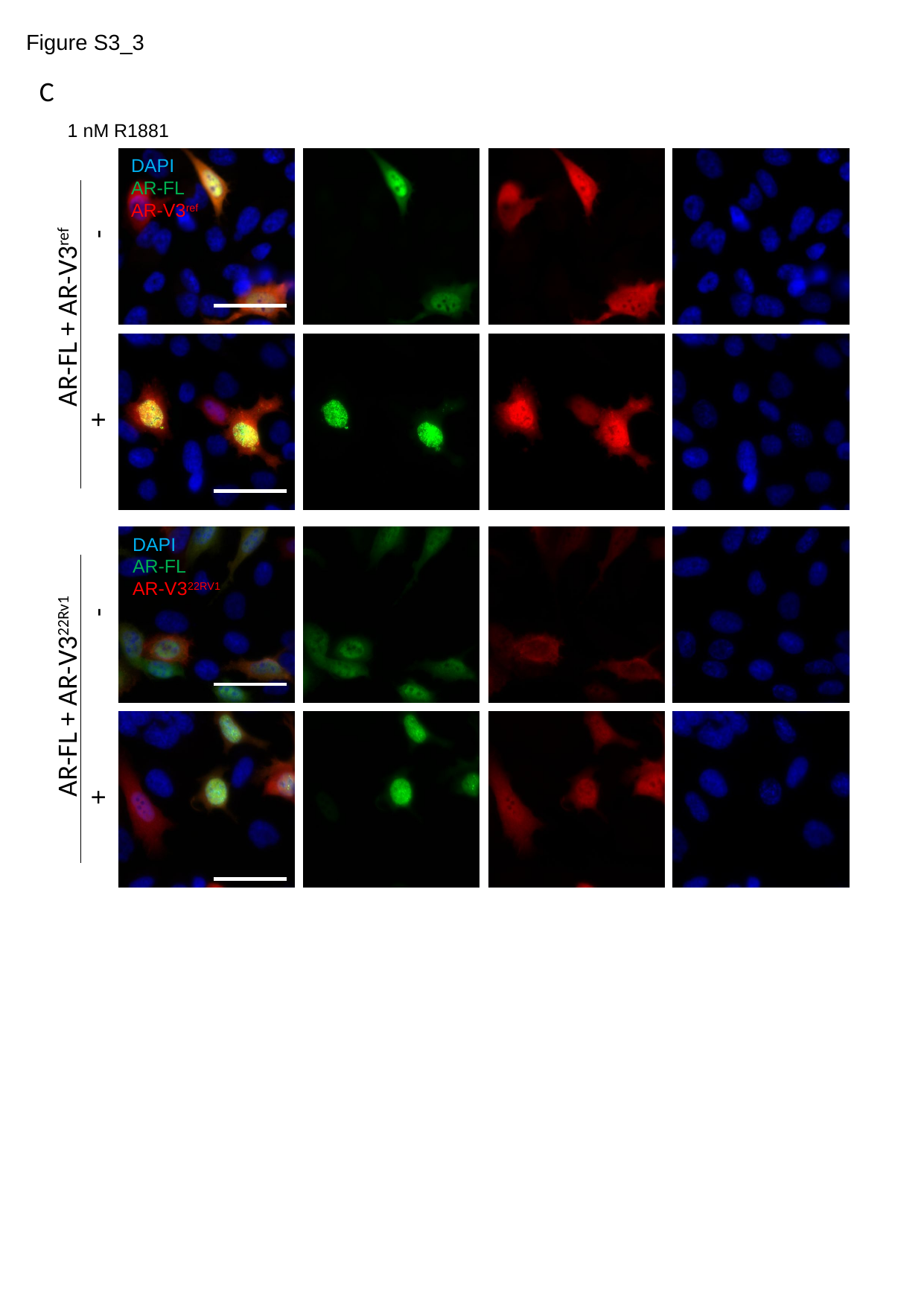

Figure S3_3
C
1 nM R1881
DAPI
AR-FL
AR-V3ref
-
AR-FL + AR-V3ref
+
DAPI
AR-FL
AR-V322RV1
-
AR-FL + AR-V322Rv1
+
