# Supplementary figures and images for: Molecular analysis of androgen receptor splice variant AR-V3 reveals eminent ambiguity regarding activity and clinical utility
Source: Cancer Cell Int. 2025 Aug 26;25:316. doi: 10.1186/s12935-025-03948-y (PMC12379386; doi:10.1186/s12935-025-03948-y)

## Slide 1
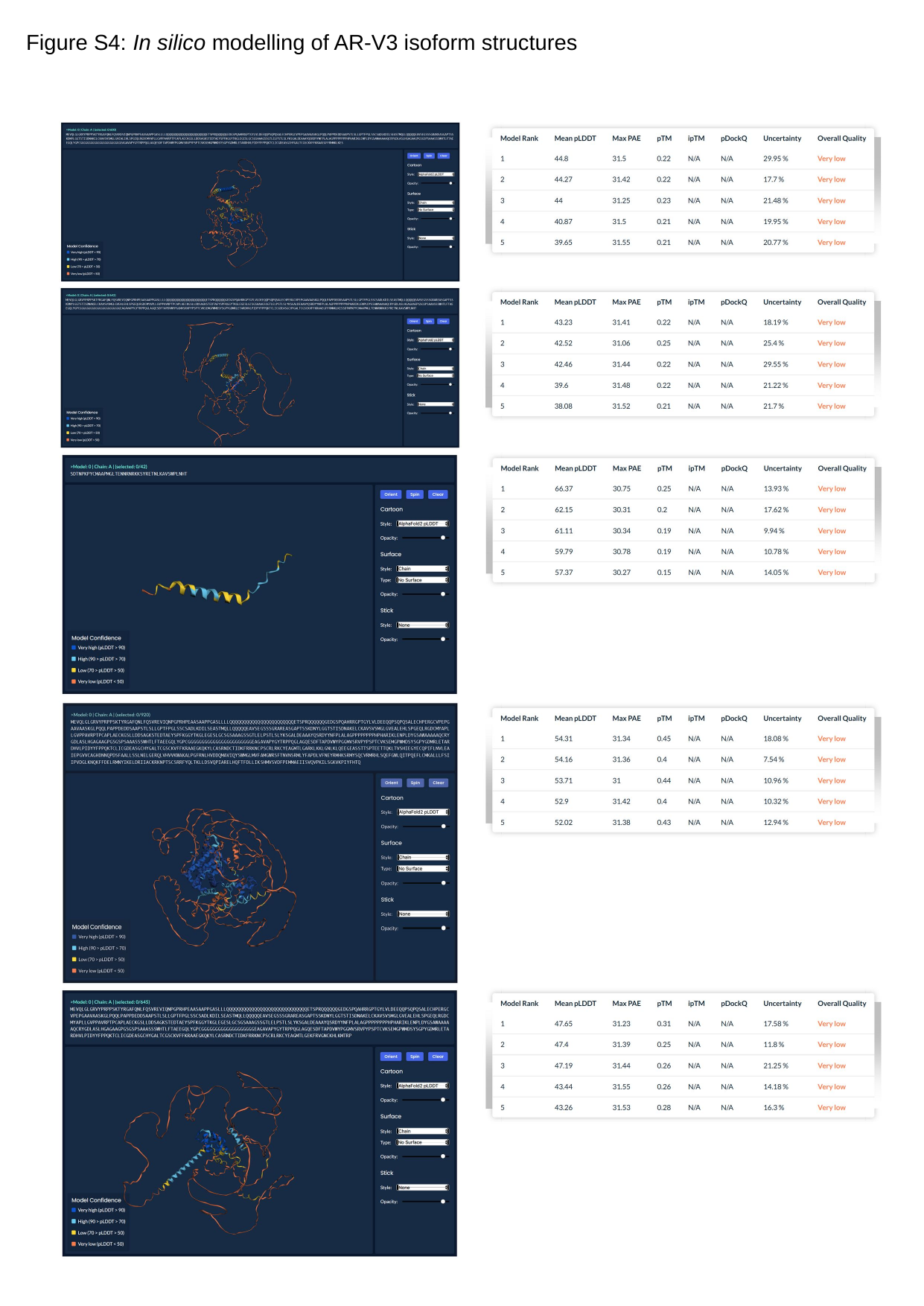

Figure S4: In silico modelling of AR-V3 isoform structures

Supplement: Supplementary file 4 — Additional file 4 [file 12935_2025_3948_MOESM4_ESM.pptx]
